# gDNA qPCR is statistically more reliable than mRNA analysis in detecting leukemic cells to monitor CML

**DOI:** 10.1038/s41419-018-0387-2

**Published:** 2018-03-02

**Authors:** Alessia Rainero, Fabrizio Angaroni, Francesca D’Avila, Andrea Conti, Cristina Pirrone, Giovanni Micheloni, Lucia Tararà, Giorgia Millefanti, Emanuela Maserati, Roberto Valli, Orietta Spinelli, Ksenija Buklijas, Anna Michelato, Rosario Casalone, Cristina Barlassina, Matteo Barcella, Silvia Sirchia, Eleonora Piscitelli, Massimo Caccia, Giovanni Porta

**Affiliations:** 1 Department of Medicine and Surgery, University of Insubria, Varese, Italy; 2 Department of Science and High Technology, University of Insubria, Como, Italy; 3 IFN (National Institute of Nuclear Physics), Como, Italy; 4 Immunology and Functional Genomics Unit, Centro Cardiologico Monzino IRCCS, via Parea 4, 20138 Milan, Italy; 5 Department of Biology and Life Sciences, University of Insubria, Varese, Italy; 6 Department of Hematology, ASST Papa Giovanni XXIII, Bergamo, Italy; 7 Department of Genetics and Cytogenetics, ASST Sette Laghi, Ospedale di Circolo Fondazione Macchi, Varese, Italy; 8 Department of Health Sciences, University of Milan, Milan, Italy; 9 Biomedical Technologies Institute, CNR, Milan, Italy

## Abstract

Chronic Myeloid Leukemia (CML) is a stem cell cancer that arises when t(9;22) translocation occurs in a hematopoietic stem cells. This event results in the expression of the BCR-ABL1 fusion gene, which codes for a constitutively active tyrosine kinase that is responsible for the transformation of a HSC into a CML stem cell, which then gives rise to a clonal myeloproliferative disease. The introduction of Tyrosine Kinase Inhibitors (TKIs) has revolutionized the management of the disease. However, these drugs do not seem to be able to eradicate the malignancy. Indeed, discontinuation trials (STIM; TWISER; DADI) for those patients who achieved a profound molecular response showed 50% relapsing within 12 months. We performed a comparative analysis on 15 CML patients and one B-ALL patient, between the standard quantitative reverse-transcriptase PCR (qRT–PCR) and our genomic DNA patient-specific quantitative PCR assay (gDNA qPCR). Here we demonstrate that gDNA qPCR is better than standard qRT–PCR in disease monitoring after an average follow-up period of 200 days. Specifically, we statistically demonstrated that DNA negativity is more reliable than RNA negativity in indicating when TKIs therapy can be safely stopped.

## Introduction

Chronic Myeloid Leukemia (CML) is a classic example of a stem cell cancer and arises when the t(9;22) translocation (the Philadelphia chromosome)^1–3^ occurs in a hematopoietic stem cell (HSC). This event results in the expression of the *BCR-ABL1* fusion gene, which codes for a constitutively active tyrosine kinase responsible for the transformation of a HSC into a CML stem cell, and thence to a clonal myeloproliferative disease.

The BCR-ABL1 fusion gene exists in three different forms, associated with different types of leukemia: p190 is mainly associated with Ph-positive (Ph+) acute lymphoblastic leukemia (ALL), p210 with 95% of CML, and p230 with a subset of patients with chronic neutrophilic leukemia (CNL). There is, however, some overlap. P210 occurs in 40% of Ph+ ALL, p190 occurs in 2–3% of CML, and p230 in some cases of CML^4^.

Until a little more than a decade ago, drug therapy for CML was limited to nonspecific agents such as busulfan, hydroxyurea, and interferon α (INF-a)^5^. Allogeneic stem cell transplantation (allo-SCT) is curative, but carries risks of morbidity and mortality; and furthermore, allo-SCT is an option only for patients with good performance status and organ functions, and who have an appropriate stem cell donor^6^. The introduction of a potent BCR-ABL1 tyrosine kinase inhibitor (TKI), Imatinib (IM), almost two decades ago, followed by subsequent generations of TKI, has transformed the management of CML^7,8^. However, these drugs are not able to eradicate the malignancy, and patients therefore require life-long therapy.

Most cases achieve a major molecular response (MMR) in which BCR-ABL1 levels detectable by qPCR (quantitative PCR) in the blood show a 3log_10_ fold reduction^9^. However, due to patient-to-patient variation, 10–20% of all patients develop even deeper molecular responses triggering dose de-escalation and discontinuation/stopping trials (STIM^10^, TWISTER^11^, DADI^12^, EURO-SKI) in which 50% of patients relapse within 12 months. TKI discontinuation studies demonstrated that stopping TKI therapy should thus be performed only under the auspices of a clinical trial^6^.

Strong evidence now shows that CML leukemic stem cells (LSCs) persist in most patients on long-term therapy, and may promote acquired TKI resistance, driving relapse or disease progression. Virtually all chronic phase (CP) patients on TKI therapy and in MMR are not cured of CML and show signs of residual disease burden from the presence of LSCs in the bone marrow (BM). Although LSCs are not always detectable in cases of very deep molecular response—most likely from technical limitations - some patients with no detectable LSCs can subsequently relapse after TKI discontinuation^13–16^. Other studies have shown that the LSCs which persist in patient in MMR express BCR-ABL1 at lower levels than the LSCs at the time of diagnosis. Furthermore, murine BM cells engineered to express low levels of BCR-ABL1 were far less sensitive to IM, whereas those expressing higher levels were prone to de novo mutations^17^. Tessa et al.^14^ demonstrated that in vitro and in vivo knockdown of BCR-ABL resulted in LSCs persistence, and these cells resulted independent of BCR-ABL kinase activity, therefore underlying that targeting BCR-ABL kinase activity alone is not sufficient to eliminate them^14^. Thus, the eradication of LSCs inevitably represent a bottleneck to cure.

Quantitative reverse-transcriptase PCR (qRT–PCR) is the most sensitive technique now available to monitor BCR-ABL1 fusion transcripts. In 2006, the National Institute of Health Consensus Group proposed an international scale (IS) to standardize the results^18^. Despite high qRT–PCR sensitivity, this technique has limits related to the interpretation of undetectable results. The mRNA molecule is susceptible to degradation, and the efficiency of cDNA synthesis can vary^19^. Indeed, the accuracy of the method critically depends on the ability of testing laboratories to measure absolute numbers of control gene transcripts in a comparable manner and achieve the sensitivity required for the BCR-ABL1 detection^20,21^. Finally, this technique detects leukemic transcripts, which may not be proportional to the number of Philadelphia positive cells; and it completely misses transcriptionally silent cells^22^.

Responding to these limitations, we have developed a gDNA patient-specific qPCR assay to detect leukemic cells irrespective of their transcriptional status, and a formula to calculate the number of leukemic cells^23^. By comparing qRT–PCR and gDNA qPCR on 15 CML patients and one B-ALL (B-cell acute lymphoblastic leukemia) patient, we statistically proved for the first time the superiority of the DNA marker to monitor leukemic cells in late follow-up (after a median of 200 days of therapy).

## Materials and methods

### Patients

All patients gave their informed consent to participate in this study.

We monitored 15 patients with CML diagnosed in Chronic Phase (CP) and one with B-ALL for an average period of 72 months (patients’ characteristics are listed in Table 1).

**Table 1:** CML patients’ characteristics

Patient	Sex	Age at diagnosis (years)	Date of Diagnosis	Diagnosis	Translocation	Start of therapy	Therapy mg/die
1	M	59	21/02/2006	CML	t(9;22)(q34;q11) p210 b2a2	07/03/2006	IM 400 mg/die
2 ^+^	M	66	06/06/2005	CML	t(9;22)(q34;q11) p210 b2a2	17/06/2005	IM 400 mg/die
3	M	60	20/05/2005	CML	t(9;22)(q34;q11) p210 b2a2	25/05/2005	IM 400 mg/die
4	M	50	30/03/2005	CML	t(9;22)(q34;q11) p210 b2a2	12/04/2005	IM 400 mg/die
5	F	71	03/02/2005	CML	t(9;22)(q34;q11) p210 b2a2	14/02/2005	IM 800 mg/die
6	F	63	02/12/2004	CML	t(9;22)(q34;q11) p210 b2a2	21/12/2004	IM 400 mg/die
7	M	52	8/10/2004	CML	t(9;22)(q34;q11) p210 b2a2	12/10/2004	IM 400 mg/die
8	M	60	30/05/2005	CML	t(9;22)(q34;q11;q24) p210 b2a2	08/06/2005	IM 400 mg/die
9	F	70	13/09/2007	CML	t(9;22)(q34;q11) p210 b2a2	24/09/2007	IM 400 mg/die
10	F	70	02/10/2007	CML	t(9;22)(q34;q11) p210 b2a2	20/10/2007	IM 400 mg/die
11^a^	M	66	03/08/2006	B-ALL	t(9;22)(q34;q11) p190	02/09/2006	IM 600 mg/die
12	M	74	12/07/2010	CML	t(9;22)(q34;q11) p210 b3a2	16/07/2010	IM 400 mg/die
13	F	20	04/08/2010	CML	t(9;22)(q34;q11) p210 b3a2	Not available	Not available
14^b^	F	49	20/08/2010	CML	t(9;22)(q34;q11) p210 b3a2	25/08/2010	IM 400 mg/die
15	M	48	02/11/2010	CML	(X;9;22)(p11;q34;q11)(ABL + BCR + ;ABL+BCR-) p210 b2a2	05/11/2010	IM 400 mg/die
16	F	67	16/12/2010	CML	t(9;22(q34;q11) p210 b3a2	21/12/2010	IM 400 mg/die

^a^Patients 2 and 11 died in 2010 and 2009, respectively

^b^BCR-ABL1 coordinates for patient 14 were taken from the derivative chromosome 9

Patients included 9 men and 7 women, with a median age at diagnosis of 49 years (range 13–67 years). 11/16 patients showed a b2a2 fusion transcript; 4/16 showed a b3a2 fusion transcript and 1/16 showed a p190 transcript. The majority of patients were treated with Imatinib (Gleevec^®^, STI571, Novartis) monotherapy at a starting dose of 400 mg/day, except for patient 5, who participated in an 800 mg/day trial and patient 11, who participated in a NILG 09/2000 protocol (ClinicalTrial.gov Identifier NCT00.58072) with a 600 mg/day IM dose. Patient 5 continued therapy with IM 800 mg/day until 64 months, when the dosage was reduced to 400 mg/day. The dose for patients 6 and 8 was increased to 600 mg/day as a consequence of suboptimal cytogenetic findings observed at 12 and 6 months, respectively. Patients 6 and 8 continued therapy with IM 600 mg/day until 73 and 44 months, respectively, after which dosage was reduced to 400 mg/day. Therapy for patient 9 was changed to Dasatinib 100 mg/day after 17 months due to skin toxicity (grade III) and then reduced to 50 mg/day because of gastrointestinal toxicity. After 47 months, patient 9 followed a dose escalation to Dasatinib 80 mg/day, which elicited a MMR. The therapy for patient 10 was changed in Nilotinib 600 mg/day at 12 months due to MMR loss, but later suspended do to cardiological toxicity and substituted with Dasatinib 100 mg/day (which elicited a MMR). Patient 11 followed a dose de-escalation to IM 400 mg/day until the transplant, followed by a dose of 200 mg/day. Patient 12 reached a stable deep molecular response (4.5 log_10_ fold reduction of BCR-ABL transcripts) and suspended the therapy after 78 months of therapy. Patient 14 interrupted IM therapy due to MMR loss after 51 months of therapy, then he switched to Dasatinib 100 mg/day and followed a dose de-escalation at 50 mg/dye Dasatinib due to diabetic nephropathy-related chronic renal failure. Patient 16 suspended IM therapy after 14 months due to severe anemia, thrombocytopenia and bowel bleeding; then he followed a restart of IM 400 mg/day. For patient 13 (pediatric at diagnosis) no information about the therapy dosage are available.

### Monitoring

ELN (European LeukemiaNet) recommends to assess and monitor the response using both conventional cytogenetics (Chromosome Banding Analysis (CBA) of at least 20 marrow cells metaphases) and real-time qPCR^21^. Cytogenetics was performed at 3, 6, and 12 months, until a complete cytogenetic response (CCyR) was achieved. Once a CCyR is achieved, CBA can be substituted by FISH (fluorescence in situ hybridization) of at least 200 blood cells nuclei. qRT–PCR was performed on total white blood cells (WBC) every 3 months. The results of qRT–PCR are expressed according to the International Scale (IS) as BCR-ABL1%^18,20,24^. BCR was used as reference gene. Once a major molecular response (BCR-ABL1 ≤0.1%^IS^) has been achieved, qRT–PCR was performed every 3–6 months, depending on baseline risk, transcripts level, and transcripts level fluctuations.

We collected bone marrow (BM) and peripheral blood (PB) samples from 15 newly diagnosed CML patients and one B-ALL patient, from the ASST Papa Giovanni XXIII (Bergamo, Italy) and from Ospedale di Circolo e Fondazione Macchi (Varese, Italy). We followed these patients for an average period of 72 months. For each patient, real-time qPCR (RTq–PCR) and gDNA qPCR^25^ were used to determine the BCR-ABL1 over BCR values on all analyzed samples. Values below detection of the qRT–PCR assay were converted to positive BCR-ABL1 values due to the logarithmic transformation, since undetectable BCR-ABL1 transcripts don’t correspond to a leukemic cells absence.

All mRNA analyses were performed following the standard operating procedures of the LabNet and GIMEMA CML Working Party.

BCR-ABL1 DNA breakpoint fusion sequences of 8/16 patients were previously characterized through a system originally developed for genome walking^25^, while the remainder 8 fusion sequences were characterized with Enrichment (Sure Select Target Enrichment – Agilent Technologies) and Next Generation Sequencing methods (HiSeq instrument, Illumina). Briefly, genomic DNA was extracted from peripheral blood or bone marrow, fragmented, ligated to adaptors and enriched in the region of interest (3 regions for BCR, corresponding to major, minor and micro breakpoint regions, and 1 for ABL). Enriched DNA was then amplified and sequenced through an HiSeq sequencing instrument. Sequencing data were processed in order to identify genomic BCR-ABL1 breakpoints coordinates in chromosome 22 and 9 for each patient. These coordinates were then used to designed a patient-specific assay, which comprises two real-time reactions: one directed against the breakpoint sequence (present in one copy only in leukemic cells), and a second directed against the BCR sequence (present in one copy in leukemic cells and in two copies in normal cells).

Informed consent was obtained in accordance with the Declaration of Helsinki principles and with approval of the Ethic Committees of Insubria University, Ospedale di Circolo e Fondazione Macchi (Varese, Italy) and ASST Papa Giovanni XXIII (Bergamo, Italy).

### Statistical analysis

Statistical correlation analysis of data was performed with Wolfram mathematica and Fortran software.

## Results

Our laboratory developed a patient-specific DNA-based assay to detect leukemic cells independent of their transcriptional status. We have previously published the results obtained by comparing our gDNA qPCR to standard qRT–PCR on eight newly diagnosed Chronic Phase CML patients; our technique resulted positive in more than 30% of samples with undetectable mRNA^22,26^.

**Fig. 1:**
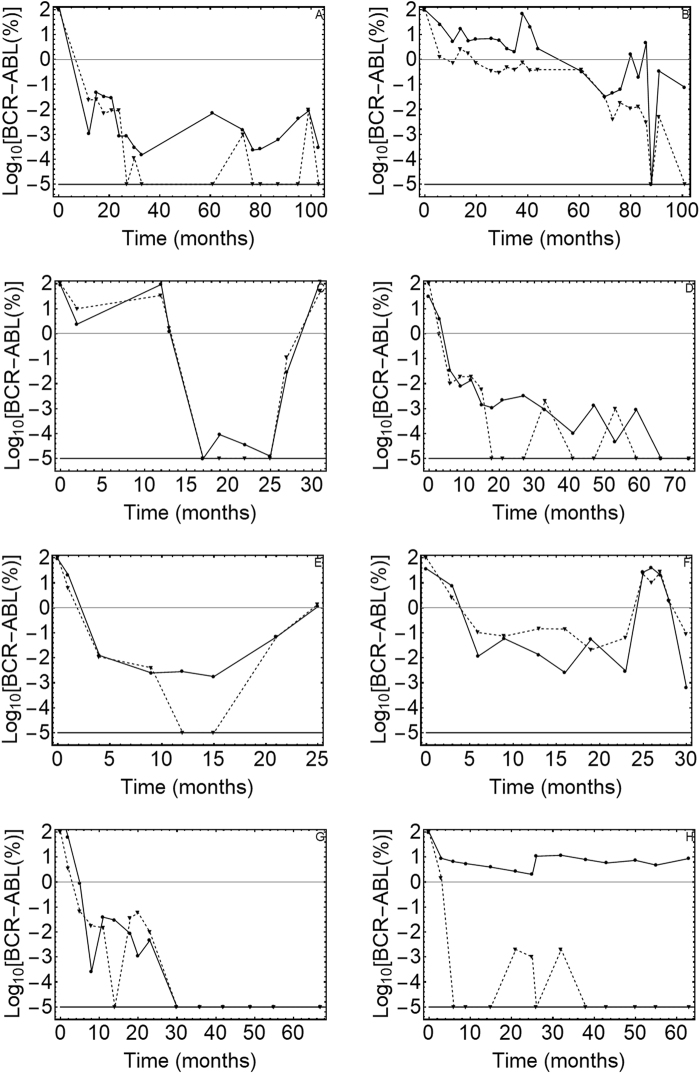
DNA vs. mRNA quantitative PCR results. The figure displays the kinetics of the leukemic cell burden in patients treated with Imatinib mesylate after the diagnosis in early chronic phase. (**a**–**h**) The panels show the percentage of cancer cells (BCR-ABL1/BCR) as measured by gDNA qPCR (⊗DNA values, solid line) and RT–qPCR (▲RNA values, dashed line) in patients 1–8

**Fig. 2:**
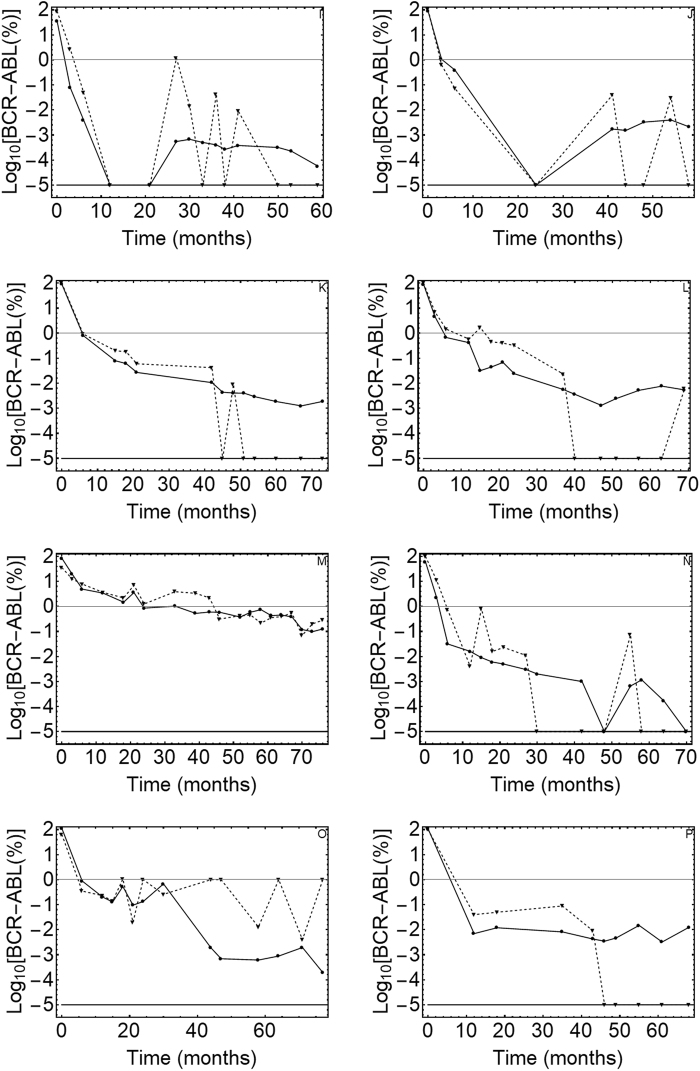
DNA vs. mRNA quantitative PCR results. The figure displays the kinetics of the leukemic cell burden in patients treated with Imatinib mesylate after the diagnosis in early chronic phase. (I-P) The panels show the percentage of cancer cells (BCR-ABL1/BCR) as measured by gDNA qPCR (⊗DNA values, solid line) and RT–qPCR (▲RNA values, dashed line) in patients 9 to 16

The results from gDNA qPCR and RT–qPCR analysis on all 16 patients are reported in Figs. 1 and 2.

Several studies^16,27–31^ based on BCR-ABL1 transcript measurements in CML patients showed a biphasic or triphasic exponential decline of leukemic cell number. The slopes of the resulting curves indicated the presence of different cell populations; the first slope represents the turnover rate of differentiated leukemic cells, while the second (and third) slope represents the turnover rate of leukemic progenitors (or stem cells). To understand the correlation between DNA and mRNA values, we first evaluated the distribution of DNA measurements after 9 months of therapy, when the number of leukemic cells is <1% (low sensitivity zone) in most of the tested patients. We hypothesized that the second slope represents the persistence of leukemic cells resistant to TKI treatment. Considering these parameters, we obtained 132 pairs of values and calculated the mean (*µ* = 2.20785), variance (*σ*^2^ = 1.0850), and skewness (*γ* = 0.16704) of these values. The skewness value suggested a symmetric distribution, and to test this hypothesis we performed a Kolmogorov-Smirnov test (implemented in Wolfram Mathematica). We confirmed the hypothesis with 95% reliability.

**Fig. 3:**
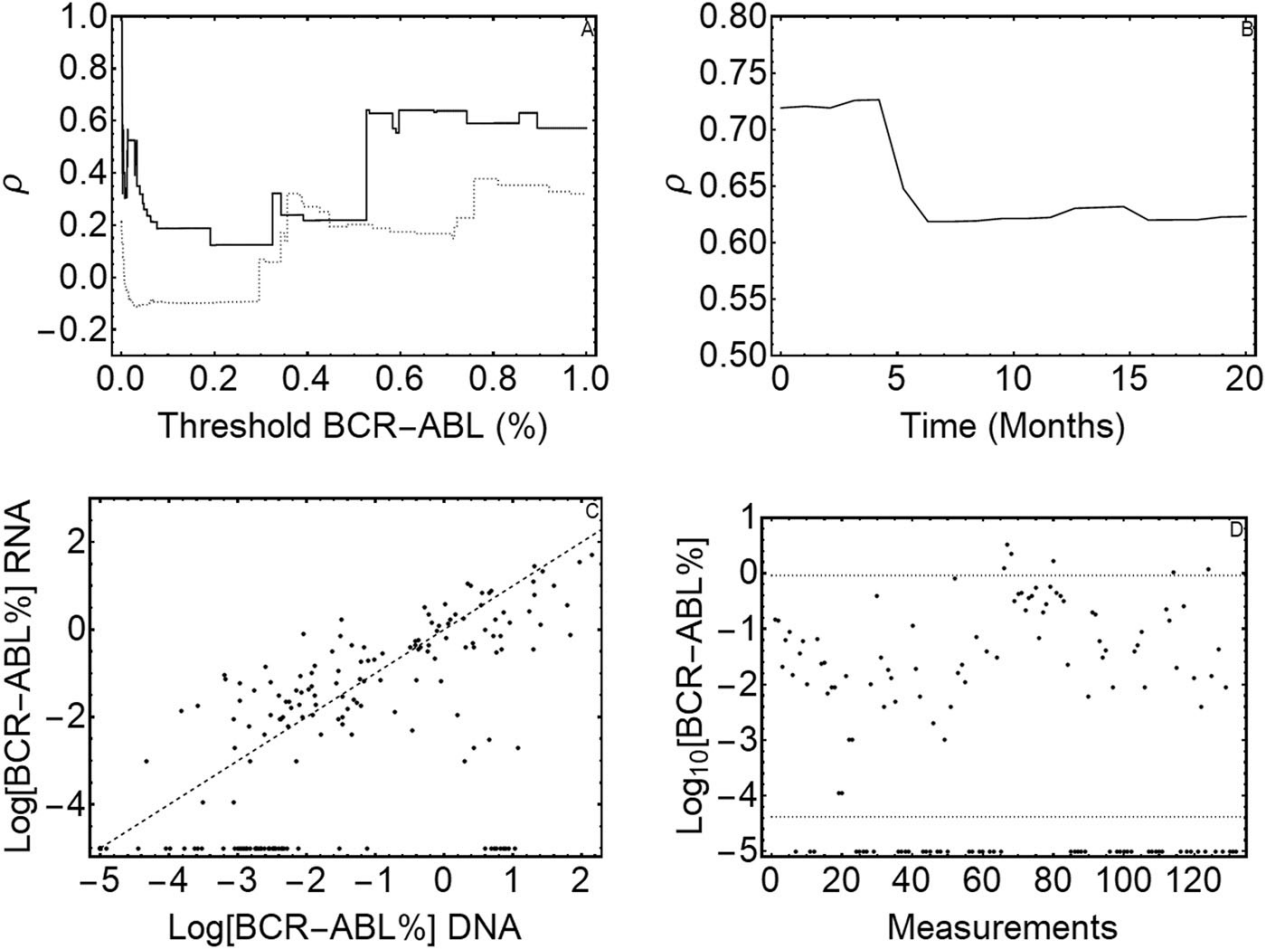
Statistical analysis between DNA and RNA measures. **a** The correlation between the two methods used for CML samples analysis. On *x* axis are reported the DNA values, while *y* axis represents the mRNA values. The dashed line is the equality line (X = Y). If there was no or little differences between DNA and RNA measures, the values should have followed the equality line. Our results are clearly not correlated. **b** The agreement between DNA and RNA values. The area comprised between the two dashed lines represents the 95% confidence interval of DNA values (not null) after 9 months of therapy. Dots represents the mRNA values coupled to DNA ones. Only 56% of mRNA values falls within the DNA confidence interval, but the 89% of measures outside this confidence interval are undetectable mRNA values. **c** The correlation between DNA and RNA values over time. On the *x* axis is the time (expressed in months), while on the *y* axis is the correlation (*ρ*) value. We observe a decrease in correlation between 100 and 200 days of therapy, which correspond to the decay of cells’ number under 1%. **d** The conditionate correlation vs. a threshold value. On *x* axis is the threshold value, while *y* axis is the correlation (*ρ*) value. The solid line represents the DNA threshold, while the dashed line represents the mRNA threshold. When we choose DNA as a threshold and we consider undetectable values, DNA and mRNA showed 100% correspondence; but we obtained a very low correlation in all the other follow-ups

To test for any gDNA and mRNA value correlation, we constructed a scatter plot (Fig. 3a) which showed no significant correlation (*r* = 0.6003).

We then tried to estimate a correlation by a different approach. First, we considered the 132 mRNA values and tested whether these were normally distributed as DNA values. The Kolmogorov-Smirnov test showed a non-Gaussian distribution of these values (*p* > 0.05). We then considered the “area of agreement” between DNA and mRNA values (Fig. 3b).

The results showed that only 56% of mRNA values fell within the DNA confidence interval, but 89% of measures outside this confidence interval were undetectable mRNA values. These results suggest that the difference between these two measures (DNA vs. RNA) might lie in undetectable values. Therefore, we studied the correlation (*ρ*) between DNA and mRNA values over time; the results within the first 10 months are shown in Fig. 2c.

We observed a decrease in correlation between 100 and 200 days of therapy, which corresponded to the decay of cell number to below 1%. Finally, we performed a conditioned correlation test (by choosing a threshold; Fig. 3d) between two arrays defined in the following way: we chose a threshold value of DNA (or mRNA) marker, then we considered the under-threshold measurements for the selected marker and the measurements made on the same sample with the other marker. For undetectable values (when using zero as threshold), because the variance is null, we used joint probability as an estimator of correlation. Results showed a low correlation between these arrays, but it is notable that DNA undetectable values were always correlated with mRNA null values; contrariwise, mRNA undetectable values have only a 21.3% probability to be correlated to DNA null values.

## Discussion and Conclusions

In most patients under TKI therapy, there is a progressive decline in BCR-ABL1 transcripts over time. However, CML has been suggested as impossible to cure, due to the persistence of leukemic stem cells that cannot be eradicated by TKI treatment. Whether all CML cells have been eradicated in any patient is a question of significant clinical and scientific importance^16^.

We have previously demonstrated that our gDNA qPCR assay proves residual disease positivity in more than 30% of mRNA negative samples^22,26^.

In this study, we performed a comparative statistical analysis of both mRNA and DNA BCR-ABL1 values in 15 CML patients (7 new and 8 previously published) and one B-ALL patient, in order to investigate if our gDNA technique is more reliable to identify transcriptionally silent leukemic cells, which could be responsible for relapse after TKIs discontinuation. As reported in the literature^32–34^, our results indicate a 3-fold decrease in BCR-ABL1 gene and transcript within the first 2 years of therapy. By plotting the Log_10_(BCR-ABL) values over time, after 10 months (i.e. after the 3log fold decay) nearly 95% of cases showed a number of leukemic cells reduced more than 99%. Considering these cases (under 1% region) we noticed a great difference in undetectable value distribution between DNA and mRNA: 17 BCR-ABL1 DNA negative samples vs. 81 mRNA negative samples. Indeed, there was weak agreement between DNA and RNA values (56%), and the decrease in correlation was consistent after the initial 3-fold decay in leukemic cell number. Our data analysis also showed that when we considered DNA as a threshold in correlation analysis, there’s always a correspondence (100% joint probability) between undetectable values of DNA with that of RNA; contrariwise, considering RNA as a threshold value, there was only a 21% joint probability (i.e., undetectable RNA values were rarely correlated to DNA null values).

We infer that after an average period of 200 days of therapy, BCR-ABL1 detection with gDNA qPCR is more reliable to determine the absence of leukemic cells and, therefore, could be more reliable for the clinicians’ decision on whether to discontinue TKI therapy.

Many researchers have proposed mathematical models to describe the differentiation hierarchy of the hematopoietic system^16,27–31^. Most of these studies were conducted on BCR-ABL transcript values from CML patients and showed a biphasic or triphasic exponential decline of leukemic cell number. The slopes of the resulting curves indicated the presence of different cell populations: the first slope represents the turnover rate of differentiated leukemic cells, while the second (and third) slope represent the turnover rate of leukemic progenitors (or stem cells). Our DNA data seem to fit with these RNA-based mathematical models, as we obtained a biphasic decline of leukemic cells for almost all our patients; however, we were not able to calculate the first slope of the curve, due to the paucity of measurements in the first 12 months of therapy.

Moreover, the molecular response at 3 months of TKI therapy has prognostic significance; a BCR-ABL1 transcript level >10%^IS^ at 3 months is associated with significantly inferior overall free survival (OS), progression-free survival (PFS), failure-free survival (FFS) and cytogenetic and molecular responses. The National Comprehensive Cancer Network (NCCN) guidelines include a change of therapy if BCR-ABL1 is >10% at 3 months, whereas ELN suggests that a single BCR-ABL1 measurement at 3 months is insufficient to define treatment failure requiring a change of therapy. For patients with >10% BCR-ABL1 at 3 months, the ELN recommends additional testing and a change of therapy for patients who are still >10% after 6 months of treatment.

In order to robustly identify the first slope, a sufficient number of early time points is needed; thus, with the aim to develop a new mathematical model to predict the effect of TKIs on leukemic stem cells, we propose that clinicians collect new CML samples every month for the first 12 months of therapy. We would also include in the resultant study CML patients who received nilotinib as first-line therapy, to understand whether this second-generation TKI is more active on the leukemic stem cell population (though Tang et al.^16^ concluded that Nilotinib elicited treatment responses very similar to Imatinib).

The application of our DNA-based method to CD34+ sorted cells (stem cells) could also provide additional information about the stem cell compartment composition in CML patients at diagnosis and during treatment.
